# De-escalation of conflict in forensic mental health inpatient settings: a Theoretical Domains Framework-informed qualitative investigation of staff and patient perspectives

**DOI:** 10.1186/s40359-022-00735-6

**Published:** 2022-02-15

**Authors:** Isobel Johnston, Owen Price, Peter McPherson, Christopher J. Armitage, Helen Brooks, Penny Bee, Karina Lovell, Cat Papastavrou Brooks

**Affiliations:** 1 School of Health Sciences, Division of Nursing, Midwifery and Social Work, Jean McFarlane Building, University of Manchester, Oxford Road, Manchester, M13 9PL UK; 2 Division of Psychiatry, University College London, 6th Floor, Maple House, 149 Tottenham Court Road, London, W1T 7NF UK; 3 Manchester Centre for Health Psychology, School of Health Sciences, University of Manchester, Oxford Road, Manchester, M13 9PL UK; 4 Population Health Sciences, Bristol Medical School, University of Bristol, Canynge Hall, 39 Whatley Road, Bristol, BS8 2PS UK; 5 Manchester University NHS Foundation Trust, Manchester Academic Health Science Centre, Manchester, M13 9WL UK; 6 NIHR Greater Manchester Patient Safety Translational Research Centre, School of Health Sciences, University of Manchester, Manchester, M13 9PL UK

**Keywords:** Aggression, Implementation science, Communication, Mental health, Nursing

## Abstract

**Background:**

Violence and other harms that result from conflict in forensic inpatient mental health settings are an international problem. De-escalation approaches for reducing conflict are recommended, yet the evidence-base for their use is limited. For the first time, the present study uses implementation science and behaviour change approaches to identify the specific organisational and individual behaviour change targets for enhanced de-escalation in low and medium secure forensic inpatient settings. The primary objective of this study was to identify and describe individual professional, cultural and system-level barriers and enablers to the implementation of de-escalation in forensic mental health inpatient settings. The secondary objective was to identify the changes in capabilities, opportunities and motivations required to enhance de-escalation behaviours in these settings.

**Methods:**

Qualitative design with data collection and analysis informed by the Theoretical Domains Framework (TDF). Two medium secure forensic mental health inpatient wards and one low secure mental health inpatient ward participated. 12 inpatients and 18 staff participated across five focus groups and one individual interview (at participant preference) guided by a semi-structured interview schedule informed by the TDF domains. Data were analysed via Framework Analysis, organised into the 14 TDF domains then coded inductively within each domain.

**Results:**

The capabilities required to enhance de-escalation comprised relationship-building, emotional regulation and improved understanding of patients. Staff opportunities for de-escalation are limited by shared beliefs within nursing teams stigmatising therapeutic intimacy in nurse-patient relationships and emotional vulnerability in staff. These beliefs may be modified by ward manager role-modelling. Increased opportunity for de-escalation may be created by increasing service user involvement in antipsychotic prescribing and modifications to the physical environment (sensory rooms and limiting restrictions on patient access to ward spaces). Staff motivation to engage in de-escalation may be increased through reducing perceptions of patient dangerousness via post-incident debriefing and advanced de-escalation planning.

**Conclusions:**

Interventions to enhance de-escalation in forensic mental health settings should enhance ward staff’s understanding of patients and modify beliefs about therapeutic boundaries which limit the quality of staff-patient relationships. The complex interactions within the capabilities-opportunities-motivation configuration our novel analysis generated, indicates that de-escalation behaviour is unlikely to be changed through knowledge and skills-based training alone. De-escalation training should be implemented with adjunct interventions targeting: collaborative antipsychotic prescribing; debriefing and de-escalation planning; modifications to the physical environment; and ward manager role-modelling of emotional vulnerability and therapeutic intimacy in nurse-patient relationships.

**Supplementary Information:**

The online version contains supplementary material available at 10.1186/s40359-022-00735-6.

## Background

Conflict events, in the context of inpatient mental health care, are defined as behaviours that threaten staff and patient safety (e.g. absconding, aggression/violence, alcohol/substance abuse, medication refusal and self-harm) [1]. Containment measures such as enhanced observation, manual restraint, rapid tranquilisation, ‘as required’ medication and seclusion, are intended to prevent or minimise harmful outcomes from conflict events [2]. However, there is a lack of evidence for their effectiveness [3] and their use carries significant risks including injury and patient deaths [4, 5], damage to staff-patient relationships [6] and excess costs [7]. Despite these harms, they continue to be used frequently [8]. Reducing both conflict and containment is therefore a high priority for patients, staff and mental health service leaders [9].

The term ‘de-escalation’ refers to a range of skills and strategies designed to reduce anxiety and distress when conflict is escalating [10] and observational evidence indicates they can eliminate the need for containment measures [11]. The specific practices involved in de-escalation have been the subject of multiple reviews of theoretical and qualitative literature [10, 12, 13]. A thematic synthesis of qualitative evidence in 2012 [10] found that de-escalation involved: emotion regulation, ensuring safe conditions for intervention, and, a range of specific strategies that were either ‘autonomy-confirming’ (e.g. offering choices, providing time and space) or ‘limit-setting’ (e.g. providing instruction or deterrents). An additional review conducted in 2013 [12] synthesised this evidence into a model which proposed de-escalation as a sequential process. De-escalation stages consisted of: ‘delimiting’ (ensuring safe conditions for intervention), ‘clarifying’ (problem identification) and ‘resolving’ (problem-solving), whilst maintaining unfailing respect, empathy and self-control. Latterly, a concept analysis involving a systematic review of theoretical and qualitative evidence [13], provided the following theoretical definition of de-escalation *a collective term for a range of interwoven staff-delivered components comprising communication, self-regulation, assessment, actions, and safety maintenance which aims to extinguish or reduce patient aggression/agitation irrespective of its cause, and improve staff-patient relationships while eliminating or minimising coercion or restriction* (p10).

Largely because of its intuitive value in reducing harms from conflict and containment, de-escalation is recommended in government policy and training guidelines internationally [14, 15]. However, a recent Cochrane review found no randomised controlled trials evaluating a specific de-escalation approach in working age adult populations [16]. De-escalation does feature in the most robustly supported interventions for reducing conflict and containment [17, 18] but only as one component of complex, multifaceted interventions. It is not possible to discern what contribution changes in de-escalation behaviour made to the positive outcomes associated with these interventions. No high-quality evidence for the effectiveness of de-escalation training exists and there is no current understanding of the specific components of training programmes that change de-escalation behaviour in practice [19].

To inform evidence-based behaviour-change interventions to enhance de-escalation, there is a need for ‘ground-up’ qualitative studies that systematically identify the factors that influence de-escalation behaviour in practice. This study uses an empirically validated model of behaviour change, The Theoretical Domains Framework [20], to qualitatively investigate mental health staff and patient perspectives on factors influencing de-escalation behaviour in adult low and medium secure forensic mental health inpatient setting. The Theoretical Domains Framework consists of 14 factors relevant to changing health professional behaviour, subsumed under three categories ‘Capabilities’ ‘Opportunities’ and Motivation. Qualitative studies informed by the TDF can generate evidence-based behaviour change targets to inform intervention development [21].

The setting was chosen because the study represents one part of the development work underpinning the EDITION programme (NIHR HTA ref: 16/101/02), which was funded by the UK’s National Institute of Health Research to develop and evaluate an evidence-based de-escalation intervention for adult acute and forensic inpatient settings. A narrative review conducted in 2012 [22], and a scoping review conducted by the authors in preparation for this paper, both confirmed that there has been no prior qualitative investigation explicitly exploring de-escalation practice in low or medium secure forensic psychiatric inpatient settings.

## Methods

### Design, aim, setting and recruitment

A qualitative design using theoretically informed data collection and analysis was adopted. The theoretical approach to the study was informed by the Theoretical Domains Framework [20], an empirically validated theory of healthcare intervention implementation. The Theoretical Domains Framework links directly to the three core constructs of the COM-B (Capabilities, Opportunities, Motivation-Behaviour) model of behaviour change [23], which enables accurate identification and development of appropriate and targeted behaviour change interventions. These theories provide a basis for qualitative investigations that can identify and describe individual professional, cultural and system-level barriers and enablers to the implementation of healthcare interventions in practice, as has been evidenced in prior investigations [21].

Our study aimed to:

- Identify and describe individual professional, cultural and system-level barriers and enablers to the implementation of de-escalation in forensic mental health inpatient settings.
- Identify the capabilities, opportunities and motivation required to enhance de-escalation behaviours in forensic mental health inpatient settings.

Two medium secure forensic mental health inpatient wards (one male one female) and one low secure (male) mental health inpatient ward in one NHS Mental Health Trust in Northern England participated. Twelve inpatients and eighteen staff from these settings participated in five focus groups and one individual interview (at participant preference). The selected Mental Health Trust was a collaboration partner on the initial funding application. Access to the wards and recruitment were facilitated by Clinical Studies Officers (CSOs) working within the Trust. CSOs liaised with ward managers of the Trust’s forensic wards to ensure their capacity to participate in the research. Once capacity was confirmed, CSOs disseminated study information packs to all eligible staff and patients on each participating ward and organised focus groups and interviews for the researchers (OP, PM) to attend.

### Participant characteristics

The method of participant selection was maximum variation sampling [24]. We sought a sample that had enough diversity to ensure we would obtain sufficient information rich cases relevant to experience of de-escalation. To this end, we monitored the sample throughout recruitment by collecting demographic and experiential data via participant questionnaire (developed by the authors for this study and available in Additional files 1 and 2). Patient questionnaires collected data including: age, sex, experience of containment interventions, diagnosis, use of illicit substances in the past 12 months (y/n), detention status (voluntary/involuntary), current admission duration and number of previous admissions. Staff questionnaires collected data including: age, sex, clinical role and experience. All staff working in the participating wards including Multi-Disciplinary Team (MDT) professionals, nurses and nursing assistants were eligible to participate. Patients were eligible if they were current inpatients in the participating wards and had capacity to consent to participate. A total of eleven in-patients (Table 1) were interviewed across two focus groups from their respective wards (male and female forensic medium secure wards) one patient from the male low secure ward was interviewed alone. A total of eighteen members of staff (Table 2) were interviewed across three focus groups based on their respective wards (female medium secure, male medium secure and male low secure).

**Table 1:** Patient sample description

Time spent as inpatient in past 12 months	Age	Sex	Ethnicity	Containment interventions experienced	Used illicit substances in past 12 months	Used illicit substances in past 12 months	MHA status	Previous admission
0–4 months: N = 1 (8.33%) 5–8 months: N = 3 (25%) 9–12 months: N = 8 (66.67%)	18–30: N = 6 31–43: N = 5	Female: N = 4 (33.33%) Male: N = 8 (66.67%)	White British: N = 7 (58.33%) Black British: N = 1 (8.33%) Mixed White and Black African: N = 1 (8.33%) Asian or Asian British Pakistani: N = 1 (8.33%) Other: N = 1 (8.33%) Not reported: N = 1 (8.33%)	Physical restraint: N = 8 (66.67%) Compulsory medication given by injection: N = 2 (16.67%) Seclusion: N = 7 (58.33%) PRN Medication: N = 7 (58.33%) Increased observation: N = 7 (58.33%) Time out: N = 8 (66.67%)	Psychotic disorders: N = 11 (91.67%) Mood disorders: N = 4 (33.33%) Anxiety disorders: N = 4 (33.33%) Personality disorders: N = 5 (41.67%) Other: N = 2 (16.67%)	Yes: N = 3 (25%)	Detained: N = 12 (100%)	0: N = 5 (41.67%) 1: N = 1 (8.33%) 2–5: N = 4 (33.33%) > 6: N = 2 (16.67%)

**Table 2:** Staff sample description

Age	Sex	Clinical role	Clinical experience
18–30: N = 2 31–43: N = 9 44–60:N = 7	Female: N = 14 Male: N = 4	Ward manager: N = 4 Senior nurse manager: N = 1 Senior clinical nurse: N = 2 Social worker: N = 1 Staff nurse: N = 5 Nursing assistant: N = 5	< 2 years: N = 4 2–5 years: N = 1 > 5–15 years: N = 7 > 15 years: N = 6

### Data collection

Focus groups were selected as the data collection method on the basis that shared discussion can lead to new insights and communal perspectives that do not always arise in individual interviews [25]. To enhance transferability of our findings, individual interviews were offered to eligible and consenting participants who were unable or preferred not to, participate in focus groups. Semi-structured interview schedules were informed by the Theoretical Domains Framework [20]. The Theoretical Domains Framework was specifically developed to identify determinants of professional behaviour change and expands into 14 domains. Four domains relate to *capabilities* required to engage in the target behaviour i.e. knowledge; skills; memory, attention and decision processes, and, behavioural regulation. Two domains relate to *opportunities* to engage in the target behaviour i.e. social influences, and, the environmental context and resources available for the behaviour to be performed. Eight domains relate to *motivation* to engage in the target behaviour i.e. social/professional role and identity; beliefs about capabilities; optimism; beliefs about consequences; intentions; goals; reinforcement, and, emotion. The topic guide to inform discussion of barriers and enablers to de-escalation was structured around the Theoretical Domains. For example, for the knowledge subdomain of the capabilities questions, participants were asked: *What knowledge do you feel a member of staff needs to use de-escalation effectively?* The topic guide was piloted with the study’s patient and public advisory panel prior to the interviews.

Questions were formulated in lay terms and definitions were provided where necessary. For example, ‘de-escalation’ was defined as *verbal and non-verbal skills or strategies to reduce unsafe behaviours such as aggression or self-harm without methods like physical restraint, medication or seclusion.* All focus groups and interviews were digitally recorded (with consent) and transcribed verbatim. Data collection continued until data saturation point was felt to be met. This was determined through discussion between the two focus group facilitators. Interviews were conducted mid-2018 and were digitally recorded. The focus groups had a duration of 1 h 01 min–1 h 12 min (M: 67 min) the interview had a duration of 38 min.

### Ethical approvals

University of Manchester institutional ethical approval to conduct the study was granted on 02/05/2018 (Ref: NHS001323) subsequent to receiving Health Research Authority (UK National Health Service) approval on 21/03/2018. The study was reviewed by South Yorkshire NHS Research Ethics Committee and favourable opinion was received on 05/03/2018 (Ref: 18/YH/0035). Participants provided written informed consent prior to participating in the study.

### Data analysis

The transcripts were analysed using Framework Analysis, which is a matrix-based method of qualitative data analysis [26]. Framework Analysis is a commonly used within qualitative health research and allows for both inductive and deductive coding [27]. The approach involves the development of analytical frameworks where individual participants or groups of participants (in the case of focus groups) (cases) are represented as spreadsheet rows and relevant theoretical concepts (codes) are represented as spreadsheet columns. The first stage of analysis (referred to as indexing) involves building an initial list of codes, informed by the theoretically important topics implicated in the interview schedule as well as by initial review of transcripts and recordings. As such, Framework analyses tend to be informed, to lesser and greater extents, by pre-existing theory. This is why the approach is used in conjunction with semi-structured rather unstructured interviews [27]. After indexing, data are charted via an exhaustive process of summarising (developing shortened summaries of relevant sections of verbatim data and imputing into relevant cells by case and code) the entire dataset. The original index and resulting analytical framework may be reshaped in latter stages of the analysis via analysis of inductive themes emerging across cases or within and across codes.

Utilising the Framework Matrices feature on the NVivo Software package data were coded deductively under each domain of the Theoretical Domains Framework [20]. Inductive analysis, within and across theoretical domains, was used to identify emerging themes describing barriers and enablers to de-escalation. Research Associates (IJ and CPB), under the supervision of an experienced qualitative researcher (OP), conducted coding. The analysts met frequently to discuss: provisional codes and their position within the framework; alternative interpretations and explanations, and, whether codes reflected the original data. Across multiple meetings, the analysts collaborated in reshaping the provisional framework, incorporating new codes that emerged at later stages and dispensing with codes that had become redundant over the course of the process. Data handling and analysis was supported with NVivo software. In the final stage, a discussion took place between the analysts (IJ, OP, CPB) to identify the most prominent theoretical domains that emerged from the analysis. Identifying most prominent domains is customary in Theoretical Domains Framework-informed analyses on the basis that this informs the development of interventions targeted at the most important factors influencing professional behaviour [28]. ‘Most prominent domains’ were identified using criteria employed in previous studies. A domain was classified as prominent on the basis of (a) the frequency that participants agreed on its importance or (b) the depth or duration of discussion of its importance [29].

## Results

**Fig. 1:**
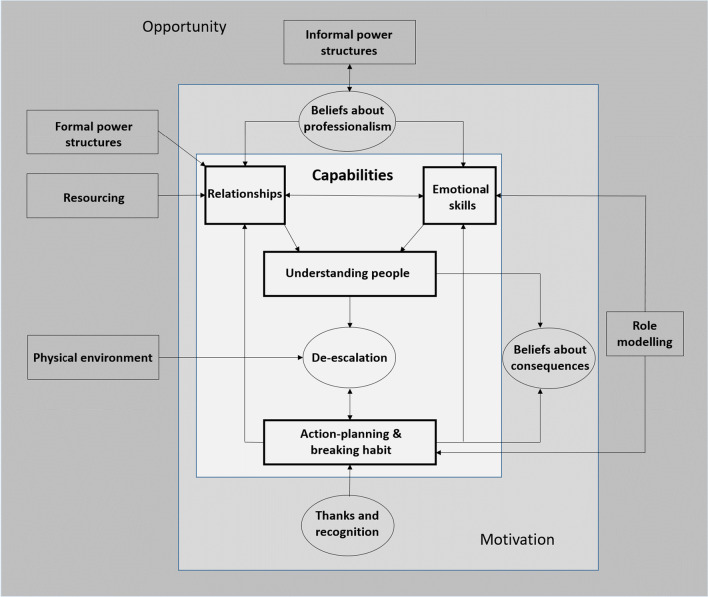
Capabilities for de-escalation

**Fig. 2:**
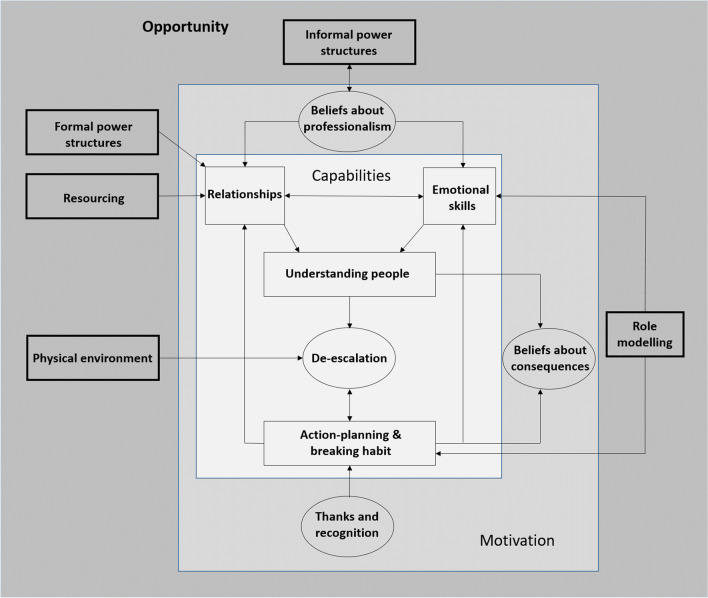
Creating opportunities for de-escalation

**Fig. 3:**
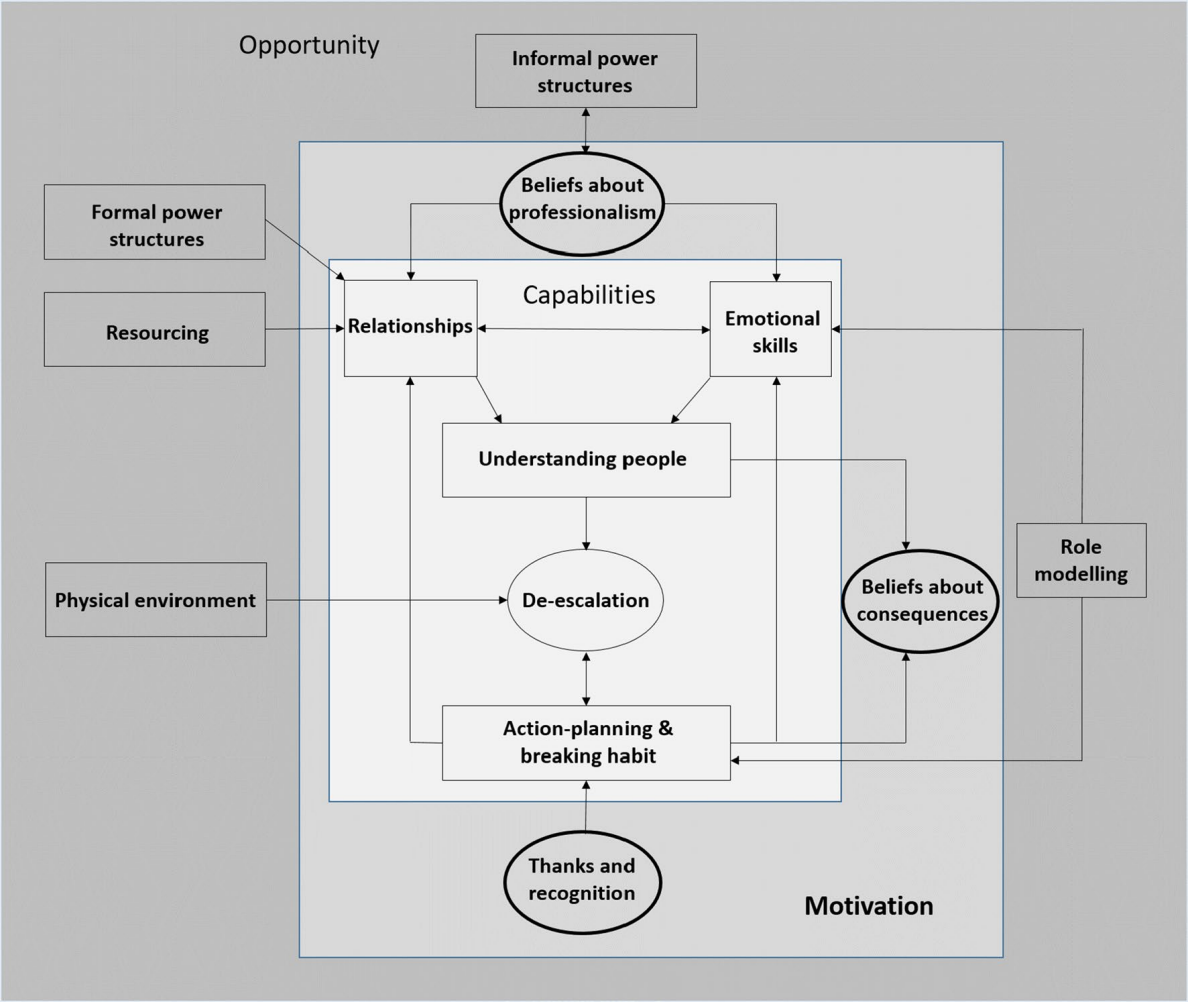
Motivation for de-escalation

There were seven prominent TDF domains identified as salient to de-escalation in low and medium secure forensic settings: psychological skills, behavioural regulation, social influences, environmental context and resources, professional role and identity, beliefs about consequences, and, reinforcement. The domains are organised in the following results according to the COM-B construct to which they pertain (Capability, Opportunity or Motivation). Whilst this structure was retained in the final framework, important themes cutting across Capability, Opportunity and Motivation categories emerged from the analysis. These crosscutting themes were vulnerability, professionalism, leadership, power and culture. The interactions across COM-B categories are explained in Figs. 1, 2 and 3 and are explored in the discussion. The following section presents a detailed analysis of the barriers and enablers to the implementation of de-escalation in low and medium secure forensic mental health inpatient settings. Differences between staff and patient perspectives are highlighted throughout the results and in the accessible overview provided in Table 3.

**Table 3:** Similarities and conflicts of staff and patients' perspectives within themes

		Capabilities
Psychological skills		
Relationship-building	**	Both staff and patients focused on investing in genuine relationships as the key facilitator. However, only staff referred to the reading of patient notes as a means of understanding patients
Emotional skills and understanding people	**	Some staff presented evidence of negative biases towards the effectiveness of de-escalation in patients with certain diagnoses (e.g. schizophrenia). Patients felt strongly this bias resulted in staff medicalising benign behaviour
Behavioural regulation		
Debriefing and collaborative de-escalation planning/	*	Only staff commented on the need for de-escalation planning and mandated debriefing to improve practise

		Opportunities
Social influences		
Formal power structures as barriers	**	Both staff and patients discussed how power imbalances in the staff-patient relationship acted as a barrier to de-escalation, with exclusion decision-making around medication being the primary example discussed. Staff also felt un-qualified staff (e.g. healthcare assistants) could be useful in diffusing conflict resulting from the power struggle between patients and nurses
Ward manager role-modelling	*	Only staff highlighted the need for support, recognition and modelling of vulnerability from ward managers
Informal power structures as barriers	**	Only staff highlighted the usefulness of the HCA role due to reduced power difference. Both groups discussed supplementary staff (e.g. bank) with patients perceiving outsiders as useful for diffusing conflict, whereas permanent staff presented a negative bias towards ‘non-regulars’
Environmental context and resources		
Physical environment	**	Claustrophobic ward environments and, interestingly, the usefulness of open access to seclusion for de-escalation was commented on by both groups
Resourcing	**	Staff spoke in length regarding how low permanent staffing numbers affecting capacity for de-escalation, and patients indirectly commented on low staffing as a barrier to staff engagement

		Motivation
Professional role and identity		
Beliefs about professionalism	**	Balancing professional boundaries with emotional presence was a key concern among staff, with some perceiving length of service to impact negatively on staff-patient relationships. Patients commented on this aspect only noting that some staff appeared to care less overtime
Beliefs about consequences		
Beliefs about safety	*	Staff presented contradictory views that more restrictive practises (e.g. seclusion) maintained the safety of the ward by removing the possibility of later escalation, but they also perceived these practises to be dangerous due to risks of injury
Reinforcement		
Thanks and recognition	**	Patients encouraged saying thanks over monetary rewards and both groups acknowledged staff feelings of being undervalued

^*^Discussed by only staff, **discussed by staff and patients

### Capabilities

Participants identified three important psychological capabilities salient to de-escalation: relationship-building; emotional skills (self-regulation and empathic attunement to the patient) and understanding the escalating person. Importantly, participants emphasised that these three capabilities were complimentary and that none operated in isolation (see Fig. 1). For example, understanding the person enabled staff to regulate fear responses. In turn, understanding the person was largely dependent on the quality of the existing relationship. Finally, the ability to develop effective relationships with patients was dependent on the individual staff’s members’ emotional capabilities (self- regulation and attunement to others). Many participants felt that understanding patients, and, its consequent effect on relationships and emotions, was enhanced and maintained by integrated systems for post-incident debriefing and collaborative de-escalation planning. The following provides an in-depth description of these capabilities.

Theoretical domain 1: Psychological skills (*An ability or proficiency acquired through practice*) [20, p13].

#### Relationship-building

The primary psychological skills relevant to de-escalation engagement were relationship-building skills. There was a broad rejection of the conceptualisation of de-escalation as a discrete set of communication skills by both patients and staff. Rather, both tended to support de-escalation as a by-product of a trusting relationship. In this sense, ‘who’ rather than ‘how’ was considered most instrumental.

*It depends on the person…There's no situation that's the same, no two situations are the same, and the way that you deal with one person will be totally wrong for somebody else. So, you need to have that relationship with that person to understand what will help*. **Staff, Male LS**

The value of staff engaging in personal disclosure and consistently meaningful interactions was emphasised by patients as key to the trusting relationships necessary for de-escalation. Patients did identify the specific value of honesty and directness in response to escalating anger as important. Patients felt that staff equivocation or avoidance of personal responsibility for bad news, was driven by the mistaken belief that this would help to avoid further conflict when, in fact, it furthered suspicion and frustration, e.g.:

*Sometimes there’s not enough staff to take you out, so you’ll look at nurse in charge and say, am I going out today? I don’t know, I don’t know, instead of just saying, yeah or no…* **Patient, Female MS**

#### Emotional skills and understanding people

Both patients and staff recognised the importance of staff remaining attuned to both their own and the patient’s emotions during incidents of potential violence. Staff identified a reciprocal relationship between vigilance to their own emotional reactions and understanding of the patient’s. This self-monitoring of emotions facilitated the use of skills felt useful to de-escalation, for example, acknowledging own vulnerabilities to stimulate empathy in the escalating patient, e.g.:

*My grandad died and they wouldn’t let me see him before he died and I went into a panic…[NAME] came and sat with me, which is one of the nurses and calmed me down by talking to me and telling me to breath, and things like that, and then she said what it were like when she lost her mum*. **Patient, Female Forensic**

However, staff identified many barriers to remaining emotionally available to patients in an environment characterised by high levels of interpersonal hostility. In order to cope, they described a process of emotional detachment or ‘desensitisation’ that increased over time. This had both positive and negative implications for de-escalation capability. It enabled staff to enter what they referred to as *autopilot mode* when aggression occurred, inhibiting normal fight or flight responses, allowing them to continue to function under stress and appear calm. However, others felt that the *autopilot mindset* blocked empathic attunement to the patient’s emotions and created a dependence on learnt behavioural scripts for managing aggression (that were not necessarily sensitive to the unique emotional contours of each potentially violent situation). They also linked psychological distress in staff with prolonged emotional suppression at work, e.g.:

*…being shouted at and threatened it's not a normal situation and it's very difficult to say, oh, I'm going to be calm because I know that's going to work better…you have to deal with it later and sometimes that might come when you're at home, when you're trying to sleep after a busy shift, and it's all there at the forefront of your mind* **Staff, Male LS**

The emotional detachment described by staff was also felt to prevent them from gaining the relational knowledge that informed whether, when and how to intervene with de-escalation. Unnecessary interventions, caused by staff misinterpreting benign behaviours (albeit loud, animated or restless) as intimidating or threatening, were felt common and unhelpful. Passive acceptance of behaviours *society would essentially deem abnormal* was felt to be a key de-escalation skill that depended on staff’s understanding of the meaning of the behaviour (and, therefore, the actual risk present) based on their knowledge of each patient. Both patients and staff felt that this knowledge was derived from staff-patient relationships that involved genuine emotional connection fostered through reciprocal personal disclosure over time. Only staff referred to the value of medical documentation as a means of gaining this knowledge.

Patients identified an important attitudinal barrier to staff gaining authentic knowledge of patients. They expressed a common view that staff were more likely to medicalise behaviour of patients with a diagnosis of psychosis and hold stigmatising beliefs that these individuals were inherently more dangerous. As a result, they felt that staff were more likely to use containment interventions rather than de-escalation with this group.

*Is there a relevance to say, right, ‘cause a person who’s given a diagnosis, and particularly one of schizophrenia, which is used for a lot of different conditions, to describe what’s going on in there, people then say, well he’s a bit schizo, he needs this.. on the assumption that a person with schizophrenia, acts in a way that’s unusual, or acts in a way that is somehow threatening*. **Patient, Male MS**

Theoretical domain 2: Behavioural regulation (*Anything aimed at managing or changing objectively observed or measured actions*) [20, p14].

#### Debriefing and collaborative de-escalation planning

Staff participants recommended collaborative de-escalation planning with patients to increase understanding of, and empathy towards, patients. They acknowledged the importance of using patient insight to inform staff approaches to de-escalation.

*The collaboration’s got to be there, hasn’t it? The service user is saying what they think they need at crisis point, rather than us just doing what we think is right, because I like to think we know what we’re doing and what we are doing is close enough to the right thing but, like I said, nobody knows the service user better than themselves. If they’re telling us at the point when they are well enough, this is what I prefer, then, you know, we’re capturing that*. **Staff, Male MS**

Recognising, however, that not all patients have insight into their own behaviour, which de-escalation techniques worked best for them, or, the necessary relationships with staff to facilitate collaborative de-escalation planning, advanced de-escalation planning was not always considered straightforward. In these circumstances, they described a process of trial-and-error to inform the development of individualised de-escalation plans, e.g.:.

*You haven't got that relationship and you've not been able to talk through because if they can't identify what works for them it's going to be trial and error until we can find something. So, we do look at PBS* (Positive Behaviour Support)… *we've got a running record of what we've tried, what works, what hasn't worked. And getting them involved*. **Staff, Female Forensic**

Post-event debriefs were repeatedly described as a *support system* for staff to aid processing of traumatic incidents. Staff made novel suggestions to include patients in debriefing to enhance understanding of the successful and unsuccessful elements of the de-escalation approaches employed, and, to feed back into de-escalation plans as a part of the trial-and-error process they recommended.

### Opportunities

Participants indicated that opportunities for de-escalation were limited by *social influences*. These related both to shared beliefs within ward nursing teams, and, more formal power structures that failed to involve patients in antipsychotic prescribing decisions. Many staff participants described common beliefs in ward teams that stigmatised emotional vulnerability in staff and therapeutic intimacy (openness and reciprocity) in staff-patient relationships. These beliefs, they felt, often prevented staff from having authentic relationships with patients, which they regarded as the most important context for successful de-escalation. The stigmatisation of emotional vulnerability in staff was perceived as a barrier to a culture of mutual support within ward teams, considered essential to enabling staff to process and subsequently regulate emotion in response to escalations of patient distress. Participants identified ward managers as instrumental in modifying staff beliefs, through role-modelling emotional vulnerability to staff, and, therapeutic intimacy in their interactions and relationships with patients. Further opportunities for de-escalation could be created through adapting the physical environment to enhance de-escalation and providing sufficient resource to minimise burnout and increase time for staff-patient relationship building (Fig. 2). The following provides an in-depth description of the opportunities that need to be created to enhance de-escalation.

Theoretical domain 3: Social influences (*Those interpersonal processes that can cause individuals to change their thoughts, feelings, or behaviours.*) [20, p14].

#### Formal power structures as barriers

Patient accounts indicated that opportunities for enhanced de-escalation could be created in response to medication-related conflict. They expressed alienation from qualified nurses who, they felt, along with medical staff, marginalised them from care decisions both in a physical sense, by making decisions in their absence, and through the use of technical language that precluded their informed participation. They felt this exclusion was most amplified in prescribing decisions where they reported having no input into decision-making. They observed that, as a result, medication-refusal often served as a means of patients reclaiming lost power.

*Talking about it and coming to some agreement on what medication they want rather than just saying, no, it’s this medication or none, do you know what I mean?* **Patient, Female MS**

Patients and staff tended to agree that healthcare assistants were more trusted and more valued by patients in the de-escalation of medication-related conflict. This was because these relationships were perceived as having less power disparity, social distance and disruption caused by paperwork. Despite this identified value, heathcare assistants broadly felt that their knowledge of patients wasn’t consulted for the purposes of de-escalation as often as it should be, either in team meetings or in ward rounds, e.g.:

*A lot of the time it's the qualified staff who go into MDT meetings, not it's…I'm a Band 3, I'm a healthcare assistant. So, even though I spend more time with service users out on ward areas my input’s not included in…even though I probably know that patient more*. **Staff, Male LS**

#### Ward manager role-modelling

Staff participants felt that the behaviour modelled by ward managers was key to creating opportunities for teams to engage in de-escalation effectively. The two key attributes for de-escalation staff felt it was important for ward managers to model were *vulnerability* and *fallibility*. Vulnerability, as has been discussed, was identified as a key de-escalation skill. To diffuse this skill amongst teams, it was felt necessary for ward managers to normalise emotional vulnerability in interactions with the ward team as well as in their interactions with patients (openness and reciprocity). This was on the basis that it would reduce the excess use of emotional suppression among nurses that was not felt useful for de-escalation.

*One of things the manager said to me, well it's perfectly fine to cry, if you want to do that now, if you go home and do it, if you turn up tomorrow morning and you just burst into tears it's normal, it's fine, but come and talk to us, or if you feel like you can't be here for a while that's perfectly normal*. **Staff, Male LS**

Fallibility was felt important to model as it provided staff with the confidence that, should de-escalation be attempted and fail, that they would not be blamed for failing to use more restrictive practices earlier. It was also more generally perceived to create an open culture in which de-escalation events could be discussed openly and honestly, e.g.:

*Staff want to see that you’re human, that you’re not just a policy and procedure person, and I think that’s important, they want to know that you’re fallible, that you do make mistakes, that it’s okay to make mistakes, as long as we all learn from them, that’s important from a management perspective*. **Staff, Male MS**

#### Informal power structures as barriers

There were difficulties identified by participants that ward managers might encounter in steering a ward team toward the two signifiers of a de-escalating team culture (vulnerability and fallibility). Participants pointed to the pivotal role of experienced staff (irrespective of grade or professional status) in influencing culture. Unusually, most participants supported an inverse relationship between experience and good de-escalation practice. One staff member stated that *staff that were deskilled were really experienced* suggesting a cumulative decline in staff competence as a result of continued exposure to modelled poor practise. This was felt a serious problem given the role of these staff in socialising new starters and junior staff to the existing culture. Perspectives indicated that team identities militating against de-escalation practice were characterised by a culture of *contempt for vulnerability*. This trait was perceived as so deeply embedded in the culture of forensic settings that staff often described a process of self-policing their emotional expression. Notably, this perception wasn’t necessarily derived from explicit statements from colleagues ridiculing emotional ‘weakness,’ but was rather more intrinsically and implicitly expressed in staff’s normative values and behaviours.

*If I went out in to car park and cried my eyes out to [DELETED NAME] it would be forgotten about the next day. You wouldn’t dare do that in here…Because it's a weakness…You'd be worried that someone would question whether you were suitable to work on the ward, whether you were suitable to deal with the situation, whether you're mentally strong enough*. **Staff, Male LS**

Participants explained that vulnerability was stigmatised in this way because it represented a threat to the appearance of a united resilience against patients. They felt this caused a resultant hostility in nursing teams to those deemed ‘outsiders’ (e.g. non-regular staff, students and even MDT professionals) who were viewed as a threat to established rules, routines and practices. Participants reported that, within these cultures, alternative perspectives on de-escalation practices, particularly when voiced by ‘outsiders’ were often dismissed as naïve. Patients, however, reported a perception that non-regular staff could engage in positive practices and behaviours with patients because they had not been acculturated to established ways of working, for example:

*Sometimes the bank staff are better, because, you know that [DELETED NATIONALITY] guy, that [inaudible 00:41:28] yeah? He’ll stop things happening. Like, there’s this lad that goes around touching everyone, and he’ll go, stop that, none of that. And 99 per cent of the other staff, don’t even say owt…* **Patient, Male MS**

Theoretical domain 4: Environmental context and resources (*Any circumstance of a person's situation or environment that discourages or encourages the development of skills and abilities, independence, social competence, and adaptive behaviour*) [20, p14].

#### Physical environment

Staff and patient accounts both indicated that increased opportunity for successful de-escalation could be created through the augmentation or modification of existing features of the physical environment. Both agreed on the usefulness of patients being able to voluntarily access seclusion rooms. Patient accounts indicated that the seclusion room, over and above side rooms, provided them with an increased feeling of protection from others and containment of impulses to harm self or others. Whilst staff agreed that some patients found this helpful, they expressed concern about this being seen by others as a form of de facto seclusion.

*He said, well what do you want me to say, do you want me to say that if you don’t shut this door I'm going to kick your fucking head in? So, we were like, yeah, okay, we’ll shut the door. And then he was like, right, I'm happy now, I feel safe, because he was getting paranoid about other members of staff, you see?* **Staff, Male LS**

Both groups agreed that increased use of sensory rooms (spaces to engage therapeutically with sensory inputs through the use of sensory equipment e.g. dimmable lights, stress balls) would be helpful, although many male patients felt that sensory interventions were patronising and variously neither age nor sex appropriate. They recommended greater individualisation of the intervention to overcome this problem, such as personalised sensory boxes (Sensory equipment chosen by patients to create individualised sensory boxes).

*It’s not really age appropriate some of the things that there is available for you to do in the (sensory) rooms, like colouring in, and things like that…they aren’t really things that grown women want to do. Some people do, but it just depends on each person’s individual…* **Patient, Female MS**

#### Resourcing

Both groups agreed that lack of staffing presented a barrier to de-escalation owing to its impact on frequency of staff-patient interactions. However, patients often observed that the staff who were available often avoided altogether, or offered only minimal engagement to, patients.

*I get on with all of them, man, I speak to everyone, but everyone rushes, they’re proper busy, it’s quite a big place, to be honest. (So you feel like the staff don’t have much time for you?: INT) Not really, I don’t think, not everyone. Sometimes. Not every time*. **Patient, Male LS**

Staff did commonly report that the change of shift durations from 7.5 to 12 h (commonly implemented across the UK health service) had resulted in a drained workforce that was less tolerant and more avoidant of patients. They felt their ability to engage with escalating patients reduced throughout a working day contributing to an increased use of containment interventions rather than de-escalation in the latter part of 12-h shifts, e.g.:

*It's quite draining on everybody. You can't do a 12 h shift with that person being as they are, you just can't do it. So, then you have to come to the decision to, we need to seclude him … you literally don’t have the energy, the physical energy*. **Staff, Male LS**

### Motivation

Participants indicated that motivation for de-escalation was limited by aspects of nursing staff’s *professional identity*. Beliefs about what constituted professional behaviour, as a member of nursing staff, limited willingness to engage in an open and reciprocal manner with patients, a relational style we labelled ‘therapeutic intimacy.’ Motivation for de-escalation was also linked with *beliefs about consequences* i.e. staff perceptions of the extent to which de-escalation could be used safely without the need for containment interventions. Post-incident debriefing was regarded as an important remedy where staff perceptions of patient dangerousness were disproportionate and/or based on misconceptions. There was general agreement amongst patients and staff that there was insufficient positive *reinforcement* of good de-escalation practice from leadership, which was considered an important motivational factor (Fig. 3).

Theoretical domain 5: Social/Professional role and identity (*A coherent set of behaviours and displayed personal qualities of an individual in a social or work setting*) [20, p13].

#### Beliefs about professionalism

Beliefs about professionalism had a significant impact on therapeutic responses to escalating aggression. Staff described a spectrum of professionalism, with perceived un-professionalism on one end and toxic “professionalism” on the other, with both acting as barriers to de-escalation. Their perspectives indicated that actual professionalism sits between these extremes, characterised by a capacity to inhibit emotional reactions (e.g. shouting) whilst remaining attuned to the emotions of the escalating patient. Staff described a struggle to balance emotional control and presence. They conveyed anxieties of being perceived as incompetent or un-professional as a result of being ‘overly’ expressive of emotion, e.g.:

*If I went out in to car park and cried my eyes out to [DELETED NAME] it would be forgotten about the next day. You wouldn’t dare do that in here…Because it's a weakness…You'd be worried that someone would question whether you were suitable to work on the ward, whether you were suitable to deal with the situation, whether you're mentally strong enough*. **Staff, Male LS**

These fears fuelled some staff’s beliefs that all emotional expression is inappropriate resulting in emotionally unresponsive and distant staff. They noted how the long-term suppression of emotions fuels resentment and subtle retaliation behaviours (e.g. refusing patient requests). Interestingly, concrete examples of un-professionalism were not provided by staff, suggesting this is significantly less of a problem than it is perceived to be. In this way, fears of being perceived as ‘unprofessional’ appear to act as a ‘bogey man’ that fuels maladaptive emotion regulation strategies and blocks authentic relationships and therapeutic responses to escalating aggression.

Theoretical domain 6: Beliefs about consequences (*Acceptance of the truth, reality, or validity about outcomes of a behaviour in a given situation*) [20, p13].

#### Beliefs about safety

There was a broad consensus among staff participants that there are circumstances in which harmful consequences would occur if de-escalation continued to be used without containment interventions. At the same time, many expressed an aversion to containment interventions on safety grounds. Although all emphasised the importance of ‘last resort’ usage of containment interventions, a consistent explanation of what ‘last resort’ meant, was not provided. Some staff explained ‘last resort’ as being the intervention used only once all other interventions had failed. Others indicated a more complex conceptualisation of last resort in which decisions to use containment may be made earlier in the escalation trajectory, based on past experience with, and, knowledge of, the patient, e.g.:

*An individual, once he gets to a certain point in his aggression, then there's just no getting through to him, you’ve just got to [seclude] for the safety of others*. **Staff,** **Male** **LS**

This indicated that the principle of last resort is highly context-dependent, influenced by intuition, experience and, potentially, also, by bias and prejudice. Whilst used liberally by staff to justify containment decisions, it is not, therefore, an especially useful linguistic expression in illuminating the actual processes involved in containment versus de-escalation decisions.

Some staff expressed the view that the presence of a seclusion room creates a dependence among staff on using this method to manage behaviour. They felt that on wards without this facility, staff tended to rely on interpersonal relationships to manage equally risky behaviour. This indicated that, for some, the belief that containment is the only available method to handle risky situations may act as an internal justification for their use without the exploration of potentially safer methods such as de-escalation.

*We don’t have exclusion areas, we don’t have those areas to take people should we need to restrain them, so interpersonal relationships, if they do escalate, we have to deescalate them otherwise we don’t have specific areas or things in place*. **Staff, Male MS**

Theoretical domain 7: Reinforcement (*Increasing the probability of a response by arranging a dependent relationship, or contingency, between the response and a given stimulus*) [20, p13].

#### Thanks and recognition

Patients and staff felt saying thanks to staff for good practice is a more effective method of reinforcing de-escalation behaviours than offering rewards. Staff repeatedly reported feeling undervalued in their work. Patients acknowledge this lack of recognition and stressed the process of giving thanks should be directed from patients, staff and management to re-instil feelings of worth among staff, fuel self-motivation and acknowledge excellence.

*It would give them that encouragement to…want to (self-achieve)… just a simple thank you that they’ve done it*. **Patient, Female MS**

## Discussion

This study aimed to describe individual professional, cultural and system-level barriers and enablers to the implementation of de-escalation in forensic mental health inpatient settings. Barriers identified at the level of individual professionals and paraprofessionals related to their capacity to regulate emotions, to understand patients, and to engage in authentic and reciprocal relationships with them. However, these capabilities were so intrinsically linked with organisational and staff team culture that their absence cannot be attributed to individual failings. Cultures that regard vulnerability as analogous with weakness and insufficiency are inconsistent with claims of a caring philosophy.

Whilst it may be understandable that attributes such as toughness and strength are valued in forensic inpatient settings where risk of physical harm is present [4], this presents a problem for de-escalation. Our analysis suggests that, in order to avoid the appearance of ‘weakness’ at work, staff spent much of their time suppressing their true emotions. They linked this with a range of negative consequences including: psychological distress; an impaired capability to be emotionally available to patients, both in their general relationships and during incidents requiring de-escalation; resentment toward patients and retaliation. These experiences are consistent with the psychological literature which broadly identifies suppression as a maladaptive emotion regulation strategy linked with: negative emotion [30, 31], reduced rapport and relationship quality [30, 32] and aggression [33].

Significantly, participants identified the value of vulnerability as an essential de-escalation capability involving the ability to (a) express and process emotions and trauma with colleagues, (b) engage emotionally with patients and (c) foster relationships with patients characterised by reciprocal disclosure and authentic interactions. There are several plausible interpretations why expression of vulnerability may facilitate de-escalation. Firstly, forensic mental health settings are characterised by extreme asymmetry between staff and patients in demands related to vulnerability. At the most fundamental level, this asymmetry is experienced in patients’ near-total dependence on staff to facilitate their basic activities of living, practically all of which are completed under the supervision and control of staff. Patients are compelled to, repeatedly, recount intimate and distressing details of their histories to members of a group who are, as our analysis suggests, socialised to regard reciprocal expressions of vulnerability as poor practice. This asymmetry is likely to be a source of shame and humiliation to patients, emotions known to be important contributors to violence in forensic populations [34, 35]. It is possible that staff expressions of vulnerability during de-escalation work because they restore the patient’s sense of dignity through an, albeit, temporary, rebalancing of power relations. Secondly, prior investigations have found that cultures that are disapproving of vulnerability are associated with poor emotion regulation [36] and emotion regulation is a core de-escalation capability [12]. Expressions of vulnerability may be generally characteristic of staff who are able to regulate their emotions in response to aggressive behaviour, in spite of social pressure imposed by the dominant team or organisational culture.

Proximal to nursing attitudes to vulnerability in staff, was the cultural stigmatisation of what we labelled ‘therapeutic intimacy’ (openness and reciprocity) in staff-patient relationships. Many staff held beliefs that anything approaching emotional closeness with patients was ‘unprofessional’ whilst others felt this belief resulted in a social and emotional distance in which, often mutual, animus and resentment were cultivated. From this perspective, this was a clear barrier to the relational context in which de-escalation is understood to work. Despite the concerns about professionalism, in our data, no concrete examples of unprofessional behaviour were provided. It is possible that staff concerns about professionalism are used as a rationalisation for avoiding emotional closeness with forensic patients. Aiyegbusi [37] attributes forensic nursing staff's avoidance of emotional connection to a fear of identification with, on the one hand, the index offences committed and, on the other, the personal histories of abuse and neglect that patients have invariably experienced. Irrespective of the underlying reason, participants clearly linked staff’s beliefs about ‘professionalism’ to reduced understanding of patients. These beliefs, therefore, represented a key barrier to staff development of the interdependent trio of de-escalation capabilities (*Emotional skills*, *relationship-building* and *understanding people*) that participants identified. We propose that interventions to enhance de-escalation in forensic settings should encourage staff reflection on the assumptions underpinning, and drivers of, the boundaries that they maintain in their relationships with patients.

System level barriers included the absence of integrated post-incident debriefing and collaborative de-escalation planning, which participants again linked with improved understanding of patients, enhanced emotional skills and improved relationships. This is in line with randomized control trial evidence that tailored de-escalation planning and post-incident debriefing reduces use of restraint and seclusion [17]. The value ascribed by participants to incorporating patient perspectives in the post-event analysis of restraint and seclusion events might emphasise the need to include ‘peer professionals’ in debriefing processes, perhaps, especially, where the involved patient is too unwell to engage in this process. Recent systematic reviews and meta-analyses indicate that peer support interventions in mental health settings have more impact on psychosocial outcomes than they do on clinical outcomes [38, 39]. This might underscore the importance of peer support, especially, in psychosocial interventions such as those aiming to enhance de-escalation and reduce conflict and containment.

A further systemic barrier related to insufficient patient involvement in prescribing. Medication refusal is a frequent precursor to the use of restraint [40], so any intervention to enhance de-escalation should address this source of conflict. Participants identified disempowerment prior to administration as the main cause of medication refusal. This indicates that interventions to enhance de-escalation of medication refusal should target prescribers. The final systemic barrier related to rules restricting patient movement within the physical environment. Our analysis indicated that interventions should seek to increase patient access, as far as possible, to all ward spaces for the purpose of de-escalation, including the voluntary use of the seclusion room.

### Strengths and limitations

The data were collected by authors OP (a mental health nurse) and PM (a clinical psychologist), which may have introduced a bias toward professionals in how the interview questions were formulated and discussed in the interviews. Both interviewers have a professional and academic interest in the reduction of containment interventions. Both therefore held the implicit assumption that containment interventions are currently used too frequently. This assumption may have introduced a bias in how the data were collected. These risks were managed by (a) the involvement of a patient and public advisory panel in the development of the interview schedules and (b) critically reflective discussion on interview facilitation between the two facilitators after each interview. Both interviewers were male and it is possible this may have altered the nature and extent of participant discussion of the sensitive topics discussed. Neither interviewer had any pre-existing relationship with any of the participants. All participants were aware of the aims of the research in terms of enhancing de-escalation (and implicitly reducing the use of containment interventions). This may have made professional participants sensitive to the potential for criticism of current practice and thereby inhibited expression of their actual thoughts, feelings and experiences. The data were analysed by authors IJ (Honorary Research Assistant and part-time nursing assistant in a medium secure forensic unit) and CPB (Research Associate with a non-forensic mental health clinical background), which may have introduced further bias towards the staff view within the analysis.

A key strength of our study was that the theoretically-informed design enabled us to identify evidence-based behaviour change targets. However, it must be acknowledged that using a more inductive methodology, e.g. Grounded Theory [41], may have captured dimensions of the phenomena that we have missed. We followed robust, previously-used criteria for identifying most prominent domains and 7 of 14 theoretical domains were classified as prominent in the final analysis. This is in line with previous qualitative studies using the Theoretical Domains Framework [28]. Nevertheless, it is possible that domains identified by our participants as having negligible relevance, may have more significance to changing de-escalation behaviour than our capabilities-opportunities-motivation configuration indicates. The diversity and extent of information rich cases included in our sample provides some confidence that this may not be the case. However, there were some limitations to the sample in this respect. The staff sample was light in male staff and non-nursing professionals. We also recruited participants from three wards across two hospitals but all in the same Mental Health Trust. Although self-selection is source of bias that is not possible to entirely circumvent in qualitative interview studies [42], it is acknowledged that many staff and patients from the participating wards elected not to participate. It is possible that these individuals may have importantly different perspectives than those who chose to participate.

All aforementioned factors may limit the transferability of our findings to some degree. Another limitation of our study is that not all themes provide a balanced input from staff and patient data. However, as our aim was to understand factors influencing professional behaviour, it is perhaps to be expected that there were some areas where patient contributions were less rich.

## Conclusions

Interventions to enhance de-escalation in in-patient forensic mental health settings should increase ward staff’s understanding of patients and modify current beliefs about professional boundaries which limit the quality of nurse-patient relationships. Our analysis indicates that changing de-escalation behaviour is likely to depend on the modification of complex interactions across the capabilities-opportunities-motivation configuration we developed (Fig. 3). These are unlikely to be optimally impacted by knowledge and skill-focused training alone. De-escalation training has traditionally focused on increasing understanding of patients and de-escalation skill development in frontline nursing staff [19]. Our findings indicate that de-escalation training in forensic settings should be implemented alongside adjunct interventions targeting: collaborative antipsychotic prescribing; debriefing and de-escalation planning; modifications to the physical environment; and ward manager role-modelling of emotional vulnerability and therapeutic intimacy in nurse-patient relationships. Future research should investigate the mental health implications of emotional suppression in forensic mental health staff. The relationship between differing emotion regulation strategies employed by staff and their de-escalation performance is an additional area of research implicated in our findings.

## Supplementary Information

**Additional file 1.** Patient demographics questionnaire.

**Additional file 2.** Clinical staff demographics questionnaire.

## Data Availability

Due to the potential for indirect patient recognition of this patient subgroup, transcripts and datasets generated during the current study are not publicly available but are available from the corresponding author upon reasonable request.

## Abbreviations

MDT: Multi-Disciplinary Team
TDF: Theoretical Domains Framework
LS: Low secure
MS: Medium secure
