# Supplementary material for: De-escalation of conflict in forensic mental health inpatient settings: a Theoretical Domains Framework-informed qualitative investigation of staff and patient perspectives
Source: BMC Psychol. 2022 Feb 15;10:30. doi: 10.1186/s40359-022-00735-6 (PMC8845398; doi:10.1186/s40359-022-00735-6)
Supplement: Supplementary file 1 — Additional file 1. Patient demographics questionnaire. [file 40359_2022_735_MOESM1_ESM.docx]

**Enhancing de-escalation techniques in adult acute and forensic units: Development and evaluation of an evidence-based training intervention. (EDITION)**

Demographics questionnaire –

Service Users

| 1 |  | What is your date of birth? |  |  |  | / |  |  | / |  |  |  |  |
| --- | --- | --- | --- | --- | --- | --- | --- | --- | --- | --- | --- | --- | --- |
|  |  |  |  | Day | |  | Month | |  | Year | | | |

| 2 |  | Are you? |  | Male |  |
| --- | --- | --- | --- | --- | --- |
|  |  |  |  | Female |  |

| 3 |  | Are you? |  | White – British |  |
| --- | --- | --- | --- | --- | --- |
|  |  |  |  | White – Irish |  |
|  |  |  |  | Any other White background |  |
|  |  |  |  | Mixed – White and Black Caribbean |  |
|  |  |  |  | Mixed – White and Black African |  |
|  |  |  |  | Black or Black British - Caribbean |  |
|  |  |  |  | Black or Black British – African |  |
|  |  |  |  | Mixed – White and Asian |  |
|  |  |  |  | Any other mixed background |  |
|  |  |  |  | Asian or Asian British - Indian |  |
|  |  |  |  | Asian or Asian British – Pakistani |  |
|  |  |  |  | Asian or Asian British – Bangladeshi |  |
|  |  |  |  | Any other Asian background |  |
|  |  |  |  | Chinese |  |
|  |  | Other, please specify here |  |  |  |

| 4 |  | | Please indicate which of the following interventions you have received | |
| --- | --- | --- | --- | --- |
|  |  |  | |  |
|  |  | Physical restraint | |  |
|  |  | Compulsory medication given by injection | |  |
|  |  | Seclusion | |  |
|  |  | PRN medication | |  |
|  |  | Increased observation | |  |
|  |  | Time out | |  |

| 5 |  | Please indicate if you have a diagnosis that would fall into the following categories | | |
| --- | --- | --- | --- | --- |
|  |  |  | |  |
|  |  | Psychotic disorders | |  |
|  |  | Mood disorders | |  |
|  |  | Anxiety disorders | |  |
|  |  | Personality disorders | |  |
|  |  | Other, please  specify here |  |  |

| 6 |  | Have you used any illicit substance in the past year? |  |
| --- | --- | --- | --- |
|  |  |  |  |
|  |  | Yes |  |
|  |  | No |  |

| 7 |  | Have you been detained under the Mental Health Act (1983) in the last year? |  |
| --- | --- | --- | --- |
|  |  |  |  |
|  |  | Yes |  |
|  |  | No |  |

| 8 |  | Please indicate how long you have spent as a mental health inpatient in the past 12 months | | | | |
| --- | --- | --- | --- | --- | --- | --- |
|  |  |  |  | Months |  | Weeks |

| 9 |  | Please indicate how many previous mental health admissions you have had | | |
| --- | --- | --- | --- | --- |
|  |  |  |  | Previous admissions |
