# Supplementary material for: De-escalation of conflict in forensic mental health inpatient settings: a Theoretical Domains Framework-informed qualitative investigation of staff and patient perspectives
Source: BMC Psychol. 2022 Feb 15;10:30. doi: 10.1186/s40359-022-00735-6 (PMC8845398; doi:10.1186/s40359-022-00735-6)
Supplement: Supplementary file 2 — Additional file 2. Clinical staff demographics questionnaire. [file 40359_2022_735_MOESM2_ESM.docx]

<Insert funder logo here>

<Insert study logo here>

<Insert NHS Trust logo here>

**Enhancing de-escalation techniques in adult acute and forensic units: Development and evaluation of an evidence-based training intervention. (EDITION)**

Demographics questionnaire –

Clinical staff

| 1 |  | What is your date of birth? |  |  |  | / |  |  | / |  |  |  |  |
| --- | --- | --- | --- | --- | --- | --- | --- | --- | --- | --- | --- | --- | --- |
|  |  |  |  | Day | |  | Month | |  | Year | | | |

| 2 |  | Are you? |  | Male |  |
| --- | --- | --- | --- | --- | --- |
|  |  |  |  | Female |  |

| 3 |  | Position |  |
| --- | --- | --- | --- |
|  |  |  |  |
|  |  | Senior nurse manager |  |
|  |  | Senior clinical nurse |  |
|  |  | Ward Manager |  |
|  |  | Team Leader |  |
|  |  | Staff Nurse |  |
|  |  | Nursing Assistant |  |
|  |  | Violence reduction specialist / PMVA trainer |  |
|  |  | Psychiatrist (SHO) |  |
|  |  | Psychiatrist (Registrar) |  |
|  |  | Psychiatrist (Consultant) |  |
|  |  | Clinical psychologist |  |
|  |  | Occupational therapist |  |
|  |  | Other (please describe) | |

| 4 |  | Clinical experience | |  |  |  |
| --- | --- | --- | --- | --- | --- | --- |
|  |  |  |  | Years |  | Months |
